# Impact of Cultivation Condition and Media Content on*Chlorella vulgaris* Composition

**DOI:** 10.15171/apb.2019.022

**Published:** 2019-06-01

**Authors:** Yunes Panahi, Ahmad Yari Khosroushahi, Amirhossein Sahebkar, Hamid Reza Heidari

**Affiliations:** ^1^Chemical Injuries Research Center, Systems Biology and Poisoning Institute, Baqiyatallah University of Medical Sciences, Tehran, Iran.; ^2^Drug Applied Research Center, Tabriz University of Medical Sciences, Tabriz, Iran.; ^3^Department of Medical Nanotechnology, Faculty of Advanced Medical Science, Tabriz University of Medical Sciences, Tabriz, Iran.; ^4^Biotechnology Research Center, Pharmaceutical Technology Institute, Mashhad University of Medical Sciences, Mashhad, Iran.; ^5^Department of Pharmaceutical Biotechnology, Faculty of Pharmacy, Tabriz University of Medical Sciences, Tabriz, Iran.

**Keywords:** Microalgae, *Chlorella vulgaris*, Nutraceuticals, Cultivation condition, Media composition

## Abstract

Microalgae are a source material in food, pharmacy, and cosmetics industries for producing various products including high-protein nutritional supplements, synthetic pharmaceuticals, and natural colors. A promising algal source for such productions is *Chlorella vulgaris* which contains a considerable protein content. Similar to other microalgae, its desirability is minimal nutrient requirements since they are unicellular, photosynthetic, and fast-growing microorganisms. Another propitious option to be produced by *C. vulgaris* is biodiesel, since it is rich in oil too. Besides, algal well thriving in presence of increased amount of carbon dioxide makes them a practicable alternative biofuel resource without some problems of the traditional ones. At the same time, *C. vulgaris* is also a promising source for nutraceuticals such as amino acids, vitamins, and antioxidants. This review aims to discuss the conditions need to be observed for achieving a favorable growth efficiency of the *C. vulgaris*, as well as targeted productions such as biomass, antioxidant, and biofuel. Additionally, different approaches to induce any specific production are also considered comprehensively.

## Introduction

Microalgae are located all around the world found in both aquatic (mostly) and all terrestrial ecosystems. They may live either individually or establish a symbiotic communication with other living organisms.^1^ Microscopic algae as photosynthetic eukaryotic microorganisms, though contains similar organelles to land-based plants such as chloroplasts and nucleus, yield more efficiently biomass. The reason is their higher performance in utilizing sunlight, CO_2_, water, etc leading to their extremely higher growth rates. Other algae advantage over plants is their ability to be cultured in various aquatic environments with less need for fertilizers and pesticides leading to less waste and pollution.^2,3^

Microalgae are being used in multiple industries including food, pharmacy, cosmetics, and environment (producing biofuels and treatment of wastewater).^4^ For example, the whole biomass of some algal species are used as protein-repository for valuable chemical agents (pigments, enzymes, additive for cosmetics) and nutritional supplements; some *Spirulina*-containing preparations are used in wound cicatrization for their therapeutical traits; *Scenedesmus*-containing preparations are suggested for treating some skin conditions (e.g. eczema); and finally some algal-originated ingredients have been addressed to suppress the HIV virus.^4-8^ Microalgae can remove the pollutants such as carcinogenic chemicals from wastewater, however, dark color and high-fat content can inhibit their growth. They can also be effectively used in the treatment of different animal farm effluents.^9,10^

The extremely high oil content of some microalgae strains has made them great candidates for producing biodiesel. Producing biodiesel from microalgae in lieu of plants (routinely sweet corn) for production subsides the concerns about reducing human food, animal feed, and other products from plants.

Despite the expensive production process, *Chlorella vulgaris* still remains of the most promising algal strains for oil production in bioreactors.^4,8,11^ *C. vulgaris* is a very resilient species of unicellular green algae classified as chlorophyta. More than half of its biomass typically consists of protein (51%-58%), and the remained consists of carbohydrate (12%-17%), lipid (14%-22%), and other different valuable nutraceuticals such as vitamins, antioxidants, and trace elements.^4^

## Microalgae cultivation

**Figure 1:**
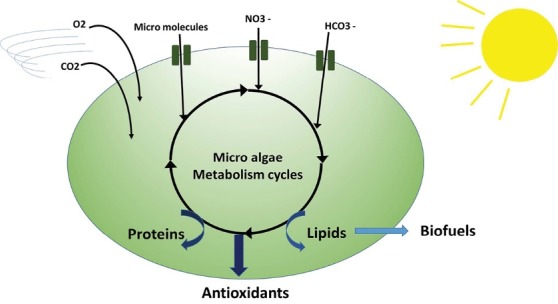
The main influential parameters on the efficiency and growth rate of *C. vulgaris* and its significant products are presented schematically in a single cell view.

Numeral arrangements have been attempted to obtain the ideal microalgal growth. To grow microalgae in the large volumes, two approaches are taken majorly: the outdoor ponds with sunlight as photo-supplier, and the outdoor or indoor photo-bioreactors (PBRs) with electric light-suppliers. Naturally, specific demands such as species control and cell growth optimization can be provided more conveniently in artificial bioreactors.^12,13^ The set-up materials, light source, circulation system through the reactor, CO_2_ supplying system, the relative amounts of different nutrients in the medium, the pH of culture media, ambient and internal temperature of the growth medium, etc. are of basic features required to be measured when designing a PBR.^12,13^ Figure 1 shows different affecting parameters on the *C. vulgaris* growth output*.*

## Process technology

Applied phycologists divide the microalgae cultivation systems into open and closed systems. In an open cultivation configuration, the culture medium is freely exposed to the ambient air under direct lighting of the sun; while, in a closed system the culture is under ceiling (indoor) isolated from the direct sunlight.^14^

### Open system

In open systems, the ambient temperature optimally should be above 15°C and continuously not less than the freezing temperature. Also, the pond’s depth should be adjusted between the light availability and the stirring optimity. The shallower bioreactors provide better light availability for algae cells, while adequate mixing requires a minimum depth. On the other hand, in shallow cultures, easy surface evaporation results in too much ionic fluctuation which is a negative factor for the algal growth.^15^ Therefore, the best dimensions of the bioreactor should be measured. Most ponds are 20-30 cm in depth. Usually, the light availability becomes a limiting factor for the cell growth typically keeping the biomass concentration between 0.1 and 0.5 g/L.

The open systems are simple to design in the forms of lakes, tanks, and various pond types including those are open, covered open, hallow artificial (raceway) ones, shallow big ponds, circular ponds, etc.^16,17^ However, majorly the three designs of circular ponds, raceway ponds, and inclined ponds are upscaled for mass cultivation of microalgae.^18^ The disadvantage of open ponds is that only few extremophile or fast-growing algae (such as *Dunaliella* and *Spirulina*) can be grown productively in such systems. Because, only these species can conquer the harsh growth conditions existing in such systems (e.g. alkaline pH) and survive contamination.^19^ However, the contamination risk in open pond systems by other microalgal, fungal, bacterial, or protozoan species is irrefutable. In spite of its less cultivation efficiency, open ponds constitute the majority microalgae production around the world because of their feasibility in setting up.

### Closed systems or photo-bioreactors (PBRs)

For the specific algal species with especial cultivation needs, fully closed systems structured from a transparent material called PBRs are better choices. PBR system provides controlled conditions such as temperature, culture density, pH levels, aeration rate, stirring program, carbon dioxide supply, water, and the light regime, around and in the culture medium. The main parameter about PBR is the best surface-to-volume ratio for highest light access in order to provide the most photosynthetic efficiency.^20^

PBRs are commonly structured as flat plates or tubular reactors in which the water and nutrients are continuously flowed into the PBR.^20^ The high surface-to-volume ratio in flat plate reactors provides the efficient use of sunlight.^21^ In these systems, narrow panels aligned in horizontal, vertical, or other angles in order to provide a uniform exposure to the light and air/CO_2_ are supplied.^20^ These systems, generally, purvey relatively condensed biomass and improved productivities.^20^ Tubular reactors are often constituted of transparent tubes in the form of parallel loops arranged vertically, horizontally, or helically,^21^ and generally do not excess one decimeter in diameter.^22^ The advantage of this system is that the culture medium is entirely separated from the environment to be preserved from the outer contaminants. CO_2_ is pumped into the medium through a longitudinal laminar flow guaranteeing enough distribution and blending in order to attain up to 6 g/L of biomass. In return, the tubes cannot be too long because the nature of water does not allow the CO_2_ or air to efficiently exchange between the environment and substrate for a long time. Then, high amounts of dissolved oxygen, sub-optimum CO_2_ supplies, and increased pH levels are remained obstacles en route of the scale-up procedure of tubular PBRs.^21^

In a comparative study on different systems of *C. vulgaris* cultivation, Lam et al reported that this algae grow most efficiently in the presence of inorganic nutrients under indoor cultivation conditions.^23^

#### Controlling of Chlorella vulgaris productions

#### Controlling physical parameters

A temperature adjustment according to the other algal species, usually suffices the *C. vulgaris* to survive. A cooling system should be considered for the temperature increase due to daylight to preserve an optimum growth rate.^22,24^ Sufficient stirring is required for good growth of algae and preventing the biomass sedimentation which is often provided by air/gas bubbling or pump-turbulating of culture medium. Mixing is especially important in reactors with a continuous light source to shift the cells from the dark to the illuminated volume for achieving a high productivity.^1^ Ideally, the growth rate of microalgae is in a direct correlation with the rate of photosynthesis. Photosynthesis rate in the PBRs depends on the amount of solar radiation that technically cannot always penetrate all throughout the culture, so that the outer layers receive more light compared to the inner volume. In addition to light concentration and distribution, its kind (sunlight or artificial light) is also important to be considered in designing PBRs. Additionally, a dark period is required to be planned for the best result.^1^

Light is defined as an electromagnetic radiation with wavelengths of between 10^-3^ and 10^-8^ meters ranging from radio waves and γ, to X-rays, and ultraviolet and visible lights. Among all, just the visible light (400-750 nm) can supply the required energy for the photosynthetic reactions.^25^ At the same time, it can also be a limiting factor if the photons either dissipate as heat or reflects instead of being absorbed. The limitation in partial absorbing photon flux density originates from the limited optical properties of the cell or the culture mixing rate. While reflection happens to a small fraction of the photons, microalgal cell density increase exponentially until any photons have remained absorbable for photosynthesis. Then, the cell growth remains constant until other limiting factors such as light density or nutrients level fall down.^1^ Another important factor in designing bioreactors for microalgae is the light periods duration (photoperiods) that importantly affect on the microalgae’s growth and photosynthesis efficiency. For example, the overexposure to light may cause an unnecessary overconsumption of electricity beside subsiding the cell growth. Whereas, a comparative study by Malaysian researchers showed a photoperiod of 12 hours (near local outdoor conditions) yielded much more microalgae biomass concentration compared to 3 h, 6 h, and 9 h photoperiods.^23^ This proportional correlation between microalgae growth rates and light period duration remains the same at different light intensities.^26,27^ Higher productivities and maximum cell density values at 12:12h (light:dark) photoperiod, in comparison with the 14:10 h (light:dark)^26^ shows that the overexposure to light reverses the results. On the other hand, a later study has shown that flashing lights could significantly increase the total fatty acid (TFA) content of *C. vulgaris* in addition to its growth rate either the lighting period was continuous or cycling (light-dark).^28^ On the other hand, Zhao and colleagues showed that red light wavelength at the intensity of 1200-1600 μmol/m^2^/s is optimum for microalgal growth.^29^ More studies regarding light impact on microalgae growth and production are summarized in Table 1.

**Table 1:** The effects of different conditions and compositions on microalgal growth for achieving the highest amount of biomass, protein, antioxidants, lipids, and carbohydrates

**Parameter**	**Mechanism/Strategy**	**Achievement**	**Ref**
**Protein and Antioxidant**			
NO_3_	More nitrate utilization and more protein accumulation	Protein content increased up to 44.3%	^30^
Traumatic acid (10^-6^-10^-5^ M)	Increase in antioxidant enzymes activity (SD, catalase, POD, GR)	The lipid peroxidation stopped by TA SH-group proteins underwent oxidative destruction	^31^
N-phenyl-2-naphthylamine (2.5 mg/L)	Inhibited photosynthesis, triggered ROS synthesis, disrupted the subcellular structure	As an allelochemical, 2.5 mg/L of it significantly increased antioxidant enzymes activities e.g. SOD, POD, and catalase	^20,32^
Nonylphenol (0.1-1.0 mg/L) exposure time	Induced oxidative stress	Obvious effects on antioxidant responses in the first day High NP contents changed the SOD, catalase, POD, and GSH levels	^20,27,33^
Elevated light intensities (400 µmol photon/ms)	Potential source of zeaxanthin^a^ So-called "molecular sunglasses" mechanism	Induced color change of microalgae from green to yellow Extra produced xanthophylls act as potential antioxidants, stabilize the membrane, and protect cells from intensive radiation	^34^
Iron-dependent oxidative stress	Triggered oxidative stress by surplus iron, decreasing the cellular growth rate of phytoplankton	>200 µM iron supply reduced the *C. vulgaris* growth level and did not changed the β-Carotene content >90 µM iron availability raised vitamin E^b^, vitamin C^b^, and total thiol content	^35^
Sodium Nitroprussiate CTAC/Flu surfactant Polycyclic aromatic hydrocarbons	SNP alleviated the pollution damage of surfactants and PAHs by providing external NO- for *C. vulgaris* cells	Supplying 20 µM SNP: Increased the biomass, the chlorophyll concentration, and the activity of SPC, SOD, POD, and catalase Decreasing MDA and ROS amounts	^20,36^
Trifloxystrobin	Decreased antioxidant enzymes’ activity Disturb photosynthesis in *C. vulgaris* Destruct the cellular structure	255.58 µg/L of trifloxystrobin (IC_50_): Reduced transcription of genes associated with the photosynthesis, soluble proteins, and T-AOC Increased SOD & POD activities and ATP expression	^37^
Azoxystrobin	AZ disrupts the *C. vulgaris* growth through: reducing energy/photosynthesis-associated mRNA expressions Inducing ROS overproduction	510 µg/L (IC_50_) of AZ: Reduced the chlorophyll and soluble protein content Increased the T-AOC level Weakened SOD, POD, GSTs, and GPx activities, and GSH content	^38,39^
**Lipid and Biomass**			
Novel lighting methods	Persistent illumination, periodical light-dark durations, persistent darkness with additional flashing light, complete darkness	The flashing light increased the growth rates of *C. vulgaris* significantly and TFA concentrations	^28^
Nitrogen and some trace elements availability	Utilized N, Mn, Ca, etc.	Lipid production reached approximately threefold	^40^
N and P content optimization	N concentration ranged 0–56 mg/L and P ranged 0–19 mg/L	N/P ratio =10: At the end of cultivation, P and N were totally eliminated and biomass concentration had reached 1.58 g/L	^41^
Light limitation Nitrogen starvation	By external shading and dimming the algal bags	Algal growth reduced due to light-limitation and nitrogen starving For future studies, separate analysis of biomass and lipid yield is suggested	^42^
Nitrogen source limitation (ammonium/nitrate)	Optimization study of sodium nitrate, ammonium bicarbonate, heptahydrate magnesium sulfate, potassium dihydrogen phosphate, dipotassium phosphate, diammonium phosphate Based on BBM and HAMGM	Optimizing the culture medium improved: Biomass: 40% (0.73 g/L) regarding BBM Lipid concentration: 85% (281 mg/L) regarding HAMGM	^43^
Harvest time optimization of chlorella growth factor	Harvest time effect of CGF extract as a growth stimulator	CGF enhanced the lipid and total biomass levels (>1 kg/m^3^) after 5 days	^44^
Temperature Nitrogen concentration Initial cell density	Temperature ranged 20-30°C	The optimal temperature for the neutral lipid productivity: 27-27.4 °C Optimal nitrogen concentration for the algal growth: 1.5 g/L Optimal cell density: 50%	^45^
CO_2_ concentration Light intensity Nitrogen deficiency Phosphorus luxury uptake	CO_2:_ 0.03, 4, 6, 12 percent Light intensity: 40, 120, 200 µmol photons/m/s) Nitrogen deficiency combined with pre-optimized phosphorus	4% CO_2_ concentration and light intensity of 200 µmol photons/m/s provided the best results Polyphosphate maximum uptake rate: 2.08 mg/L/day Better performance under nitrogen deficiency than nitrogen sufficiency	^46^
MgSO_4_·7H_2_O, KNO_3,_ Glucose, and NaCl	Using the Plackett–Burman design to selected the key nutrient factors Applying the Box–Behnken design to optimize the strategy	Maximum predicted biomass concentration (4.28 g/L) was verified with these calculated amounts: glucose (25 g/L), MgSO_4_·7H_2_O (1.33 g/L), KNO_3_ (1.30 g/L)_,_ NaCl (3.02 g/L)	^47^
Glucose, acetate, and glycerol	Autotrophic, heterotrophic, and mixotrophic modes	The heterotrophic nutrition mode yielded the maximum biomass (8.9 g/L) and lipid contents (36.19%) in CM	^48^
Red light intensity	Optimization Red light intensities: 800, 1200, 1600, and 2000 µmol/m^2^/s	The best visible light for the optimized microalgae growth was red wavelength The optimal concentration of light was 1200-1600 µmol/m^2^/s	^29^
Inorganic carbon, phosphorous Light intensity	Chemostat cultivation	Maximum biomass (538 mg/L/d) and lipid (128 mg/L/d) contents achieved at the first phase	^49^
Phytohormones	Auxins and gibberellins range 40-60 µM Microalgae spp: *Scenedemus abundans*, *Chlorella ellipsoidea*	Phytohormones promoted the microalgae due to reducing the intracellular ROS level >3-fold increment for *S. abundans* ~7-fold increment for *C. ellipsoidea*	^50^
Blue green-11 medium, Bold basal medium, Fog's medium, and Basal medium	Comparison screening Optimization	BG-11 yielded higher lipid content A2:1 mixture of chloroform:methanol was identified as the most effective ratio, extracting an average of 15% total lipids	^51^
Nitrogen sources: Urea, KNO_3_, NaNO_3_, and NH_4_NO_3_	Optimization Microalgae Sp.: *Chlorella sorokiniana* Urea concentrations: 0-10 g/L	1.5g/L urea purveyed the highest biomass concentration (0.220 g/L) and 61.52% lipid content	^52^
Light condition light/dark photoperiod	Optimization	Suitable light intensity: 2000Lux Appropriate light-dark photoperiod: 12:12	^53^
Sodium Erythorbate as common antioxidant	NaE range: 2.0-16.0 g/L Moderate controlling the accretion of the dissolved photosynthetic oxygen in the media	Algal autotrophic growth enhanced effectively The NaE treatment provided higher pigment contents (4.17 to 4.44 times), cell density (2.67 times), and algal biomass (1.21 times) compared to the glucose treatment	^54^
**Lipids and Carbohydrates**			
Light concentration CO_2_ level Stirring	Hybrid bioreactors benefiting the features of both pneumatic bioreactors and stirred tanks	Microalgae grew exponentially for around one week Entered the stationary phase after 9-14 days	^55^
Carbon sources: Glucose Glycerol Acetate	Glucose optimization using mixotrophic cultivation Assessing the usability of wastewater for renewable biomass and high-value microalgal oil production	Appropriate carbon source concentration: 5 g/L glucose Optimized biomass yield: 0.13 g/L/d Dry cell weight: 1.39 g/L Total lipid content: 19.29 ± 1.83% Total carbohydrate: 41.4 ± 1.46%	^56^
Glucose Nitrate Phosphate	Artificial neural networks A feed-forward method Optimization	The optimized medium condition: glucose (15 g/L), N (1.04 g/L), P (0.005 g/L) The highest lipid yield: 1.944 g/L The highest biomass yield: 0.31 g/L/d	^57^
Light	Supplying glucose substrate for the oil-rich microalga *Chlorella zofingiensis*	Light attenuates lipid accumulation possibly by inhibiting lipid biosynthetic pathway and promotes proliferating the cells and starch synthesis Attenuated lipid accumulation caused carbon transform from glucose to starch	^58^

Abbreviations; SD: sodium dismutase, TA: Traumatic acid, POD: ascorbate/NADH peroxidase, GR: glutathione reductase, ROS: reactive oxygen species, SOD: superoxide dismutase, NP: Nonylphenol, GSH: glutathione, SNP: Sodium Nitroprussiate, PAHs: polycyclic aromatic hydrocarbons, SPC: soluble protein content, MDA: malondialdehyde, T-AOC: total antioxidant contents, AZ: Azoxystrobin, GSTs: glutathione S-transferase, GPx: glutathione peroxidase, TFA: total fatty acid, BBM: Bold’s Basal Medium, HAMGM: Highly Assimilable Minimal Growth Medium, CGF: chlorella growth factor, CM: culture medium, BG-11: Blue green-11, medium, NaE: Sodium Erythorbate. ^a^ A macular pigment that protect eyes against age-associated macular degeneration ^b^ Vitamin E: α-tocopherol, vitamin C: ascorbate.

The biomass concentration is also under the influence of initial culture pH parameter. High pH level can reduce the growth rate and the lipid output of some microalgal species, however, extremely higher pH can precipitate calcium salts.^4,59^ According to the reports, different strains of *C. vulgaris* tolerate low pH levels (from 3.0)^60,61^ better than high levels of pH (i.e. 11).^59^ In this regard, pH=2 stops *C. vulgaris* growth completely, pH=4 leads in reduced cell number, while pH=6 cause that *C. vulgaris* grow faster than in pH=8 (however, they yield similar final cell number), and higher pH (10 and 12) reduce growth even more. Then, apparently, pH=6 is the approximate desired pH for *Chlorella* cultivation.^62^

#### Controlling media components

The growth level as well as the final concentration of microalgae are highly influenced by media composition.^61^ Nutrient-rich (eutrophic) waters lead to more microalgal blooms.^63^ Providing all essential nutrients in the growth medium of algae is necessary for adequate biomass production.^24^ The first and the most important ingredient that contributes to the algal production is carbon. Organic sources of carbon such as acetic acid or peptone as well as inorganic from of carbon dioxide can be supplies in the medium.^1^ Nitrogen is the next essential nutrient involved in algal biomass production usually added to the medium in the nitrate (NO_3_-) form.^1,64^ Nitrogen consists more than 10% of the biomass content and its shortage in culture medium leads to decreasing the chlorophylls, increasing carotenoids, accumulation of polysaccharides and certain oils,^1^ and increasing lipid production.^64^ Nitrogen deprivation gradually reform the lipid constituents from free fatty acids-rich compositions to triglyceride-rich lipids.^64^ The third essential element in the medium for the cellular processes involved in the growth such as DNA anabolism and energy transmission, is phosphorus. It is mostly added in the form of orthophosphate (PO_4_^-3^).^1^ Other elements involving selenium, potassium, sodium, iron, magnesium, and calcium plus the trace elements such as boron, copper, manganese, zinc, and molybdenum are other important nutrients mostly used in enzyme reactions.^1^ In general, the water used for preparing the cultivation systems are recommended to be distilled, filtered, or de-ionized. However, the groundwater or any other available domestic aqua is acceptable for making large-scale growth medium.^1^

Although the best conformation of culture medium for growing *C. vulgaris* has remained controversial, blue green-11 (BG-11) has been the most common medium of choice.^65-67^ This culture medium mostly consists of NaNO_3_ and K_2_HPO_4_^68^ as supplying resources of nitrogen and phosphorus. However, other culture media providing enough nitrogen and phosphorus are also frequent.^64,69^

There are other factors which should be specifically controlled during algae cultivation. The biggest is the saline concentration of the culture media. Excessive amounts of salt can inflict the growth and metabolism of algae cells and reduce the growth of *C. vulgaris.*^70,71^ Less than four decades ago, the positive effects of little amounts of NaHCO_3_ and bubbling with CO_2_ enriched air on the *C. vulgaris* have been approved.^72^ For example, a concentration of 3.36 g/L of NaHCO_3_ could yield 0.67 g/L of dried biomass.^73^ Recently, it has also been shown that ammonium in the form of NH_4_Cl is firstly consumed by *C. vulgaris* before it goes for the nitrate present in the inoculum.^6^ For different strains of *Chlorella*, a trace amount of nitrates has been enough for obtaining adequate biomass and lipid concentration, so that even 1.24 g/L KNO_3_ sufficiently nurture the cultivation.^74^ A comparison of cultivating *Chlorella sorokiniana* in presence of either 15 g/L of ammonium chloride or 1.5 g/L of sodium nitrate, resulted in same biomass concentration (both in Tris-Acetate-Phosphate media bubbled with 5% CO_2_ enriched air). However, in the NH_4_Cl-containing culture medium, the pH decreased more radically.^75^ Adding sodium bicarbonate to the basal media in a range of 0.1-1.6 g/L, showed that maximum biomass production (0.6 g/L) of *C. vulgaris* takes place at the 1.2 g/L concentration of sodium bicarbonate.^59^ Also, fixation of dissolved CO_2_ in the bicarbonate form using NaOH instead of adding CO_2_ alone, has led to more increases the final harvested biomass of *Chlorella* spp.^76^

Beside the total biomass, the production level of other microalgae’s constituents such as proteins, lipid, carbohydrates, and antioxidants is also under the influence of media content. For instance, Xie and colleagues showed that after a deprivation period, *C. vulgaris* consumed more nitrate and produced more proteins up to 44.3%.^30^ In an optimization study, a combination of nitrogen, magnesium, calcium, and some other trace elements provided a synergistic multi-parameter boosting effect and tripled the lipid production.^40^ Adding NaHCO_3_ to the media along with bubbling with CO_2_ enriched air also increases the lipid production of some microalgal species. Maximum concentration of lipid for *Chlorella* have been shown to happen at the 75 mg/L concentration of NaHCO_3_ with 4758 ppm of CO_2_. In addition, a glucose concentration of 5 g/L in *C. vulgaris* culture medium significantly increased the ultimate harvested biomass containing higher amounts of lipid, carbohydrate, and proteins.^77^

Furthermore, microalgae are addressed as source of nutraceutical antioxidants due to their rich content of carotenoids, vitamins, and phenolics. Various studies have aimed maximizing the yield of antioxidants by induction of different stress factors. As an example, Sun et al showed that trace amounts of selenium (<75 mg L^-1^) can increase *C. vulgaris* cell growth rate, organic selenium content, and antioxidant activity through increasing production of some photosynthesis associated pigments and heme containing enzymes such as guaiacol peroxidase (GPx), catalase (CAT), and superoxide dismutase (SOD). However, overexposure to the selenium (>100 mg/L) can reverse these changes.^78^ More elaboration on the antioxidant production by microalgae and important affecting factors will be discussed in following section. Besides, details of the best condition for microalgae total lipid content in regards to biofuel production will be reviewed later.

The highest amounts of *C. vulgaris* biomass, and its protein, antioxidant, lipid, and carbohydrates contents achieved through variation in cultivation conditions and media formulations are summarized in Table 1. The studies are categorized based on the main affected production of *C. vulgaris* and some other species of *Chlorella*.

#### Induction of antioxidant production in C. vulgaris

**Figure 2:**
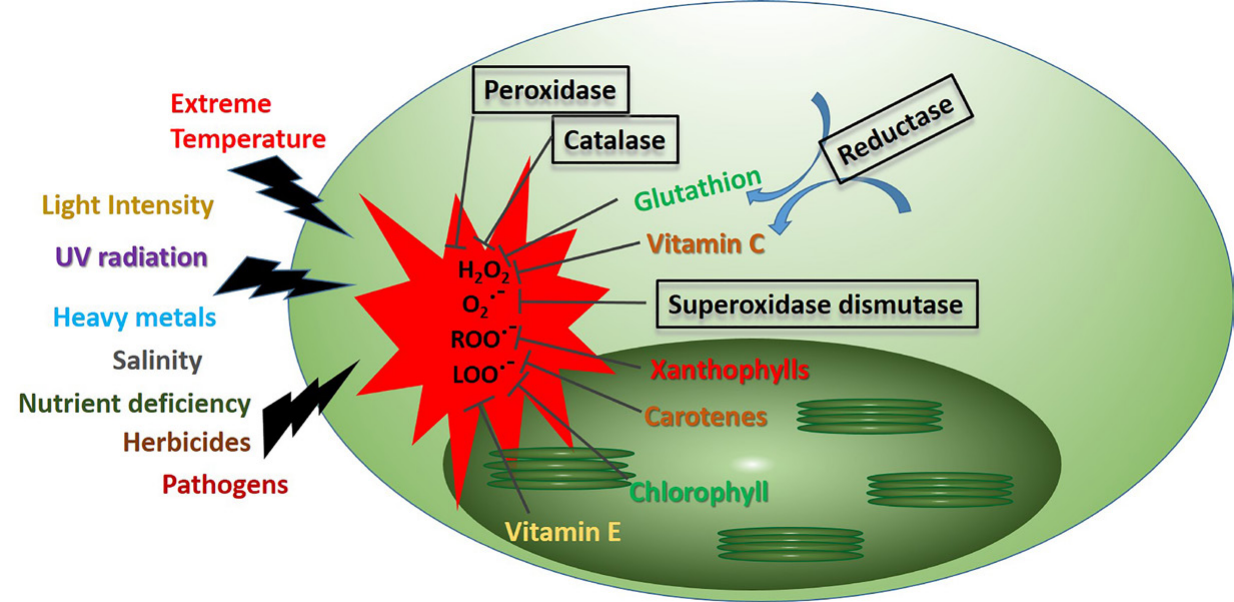
The most important stressful factors which induce oxidant and following antioxidant level of microalgae presented schematically in a single cell view.

In the general metabolism process of green microalgae, like *C. vulgaris*, oxidant reactive molecules such as H_2_O_2_, free radicals derived from molecular oxygen or chemically active oxygen species (ROS), and oxidized lipid derivatives produced at chloroplast, mitochondria and peroxisomes compartments which is reviewed before.^79^ Meanwhile, these oxidizing agents were scavenged and deactivated through adapted Enzymatic and Non-Enzymatic antioxidant pathways (Figure 2).

Normally, there is a balance between production of oxidant and antioxidant molecules inside the cell; as if there was an augment in oxidizing agent production due to some stressful conditions, cell prevents the hazardous effect of accumulated oxidizing agents by overproducing antioxidant molecule.^80^ Accordingly, numerous strategies have been adopted, in which by providing the artificial stressful cultivation condition, microalgae antioxidant machinery have been boosted up.^81^ We will discuss light intensity, temperature, osmotic stress and nutrient limitation as main approaches to enhance antioxidant production in the following sections.

##### Light

Light intensity considered as one of the most important antioxidant production inducer. While electron transfer apparatus in photosystems has been saturated, or NADP supply of the cell has been limited, photo-inhibition occurred and the excitation energy transferred to oxygen and produce reactive oxygen derivatives.^82^ In this stressful condition several adaptation mechanisms have been taken, which mainly include: 1) reducing the antenna size, 2) dissipating the energy as heat, 3) CO_2_ fixation in the forms of storage materials such as starch and lipids to redeem the reductive pressure of the electron transport chain, and 4) quenching ROS by antioxidant production.^81^ The photo-inhibition and oxidants overproduction enhance while microalgae are under the stressful growth limiting condition due to nutrient limitation or other unfavorable cultivation condition. The light stress can also promote algae to produce antioxidants including carotenoids, some vitamins, and butylated hydroxytoluene (BHT).

As an example, 4.4 kJ/mdose of UV-B exposure was taken as a stressful condition in a study to increase antioxidant value of *C. vulgaris*. This approach significantly increased radical content in the membrane as well as the superoxide dismutase activity by 40% and that of catalase by 500%.^83^ Moreover, Grudzinski and colleagues showed that intense lightening (400 μmol photons/m/s) induced yellowing of green microalgae, over-synthesizing xanthophylls. Xanthophyll is a carotenoid with potential to act as antioxidant, stabilize the membrane and, specially, protect cells from severe radiation, via “molecular sunglasses” mechanism.^34^

##### Temperature

Low temperature is one of the methods to increase antioxidant level in microalgae. Algae produce unsaturated fatty acids adding them to the membranes to sustain its fluidity in order to conform with the cold environment. Other adaptation mechanisms contain a) producing cold shock and antifreeze proteins, b) producing more enzymes to counterbalance the poor specific activity of enzyme,^84^ and c) involving differential energy partitioning.

Moreover, in higher temperature there are some evidences of increased production of astaxanthin,^85^ unsaturated fatty acids, ice structuring proteins, and phenols in some microalgae species. Miranda et al showed almost doubling the antioxidant activity of *C. vulgaris* culture at 30°C. This is possibly due to the increase of different phenolic compounds in the methanolic extract.^86^

##### Osmotic stress

Variation in salinity is one of the important reasons of osmotic changes which can impose hypo- or hyperosmotic tension on the microalgae cells. Osmotic adaptation involves (a) water flux, (b) ion transport, (c) producing vacuoles to isolate the redundant ions,^87^ and (d) producing one or several organic osmolytes such as glycerol.^88,89^

Salinity tolerance depends on environmental conditions such as light and temperature as well as nutrient constraint. Hyper-salinity sometimes increase the production of the metabolites (e.g. palmitic and oleic acids), as well as carotenoids (e.g. lutein, β-carotene),^90^ or astaxanthin.^88^ In the result of osmotic stress, valuable metabolites including glycerol, unsaturated fatty acids, and carotenoids may also increase in the microalgae cultivation. Pandit and colleagues in a recent study evaluated salinity stress (0.06 to 0.4 M NaCl) on *C. vulgaris* biomass and its productions. Their results confirmed salt tension as a significant stress that not only cause a fatty acid production enhancement and improve their composition, but also augment the antioxidant production level of *C. vulgaris.*^91^

##### Nutrient limitation

Deprivation of any major nutrients leads to the cessation of microalgae growth cycle and changing other cell metabolites output. Thus, the microalgae takes different adaptation mechanisms including a) up-regulation of enzymes associated with the mechanisms of limiting nutrient absorption; b) decrease in photosynthetic activity; c) oxidative stress following to Calvin cycle inhibition; d) producing large amounts of carotenoids;^92^ e) significant increase of protective pigments such as astaxanthin mainly via key nutrient deficiency (e.g. N, P, or S deprivation)^93^; f) increase cellular content of lipids (mostly taken as a response to nitrogen starvation),^94^ and g) upgrading the amount of polyunsaturated fatty acid.^95^

Therefore, heightening the producing level of secondary carotenoids, polyunsaturated fatty acid, and vitamin E^96^ as important antioxidants can be induced by nutrient limitation in microalgae. In the same regard, specifically in *C. vulgaris*, lipid production efficiency is mostly induced by the sulfur deprivation. Sakarika et al achieved a high level of total lipid (53.43 ± 3.93% gram per gram dry weight) in a kinetic study.^97^

#### Induction of Biofuel production in C. vulgaris

Exponentially reduction of non-renewable energy resources has propounded microalgae as an alternative source for producing biodiesel. The sustainable photosynthetic efficiency of microalgae plus the eco-friendly approach of algal petrodiesel generation have promoted their potential. Also, the non-arable land is utilized for biodiesel production from algae which appease the food versus fuel dilemma. Biodiesel is considered as a non-toxic and sustainable fuel because of its biologic origin and degradability. Additionally, the gaseous pollutants emitted by biodiesel is confirmed to be less than fossil diesel and it does not show negative effect on the carbon dioxide or sulfur amounts of the atmosphere.^64^ Furthermore, microalgae cultivation for biofuel production can be conjoined with wastewater which is reasonable and helpful in terms of economy and environment conservation. Using algae for biofuel production is a sustainable approach, since during which lots of water and nutrient (e.g. N and P elements) are recovered.^4^

Before, there have been some controversy on the successfulness of biofuels as sustainable alternative for energy resources. Because, biofuels were firstly generated from agricultural edible products (including sugarcane, sugar beet, and corn) that primarily overlapped with the staple food of people. Then, one would discuss that world’s food and water markets could be at risk and it also could compromise the forests. Next, biofuels were suggested to be produced from lignocellulosic substrates reminded from agriculture and wood industries whose converting technology to liquid biodiesel is not commercially productizing enough. The third generation biofuels seem to be technically sustainable resolving many of previously addressed problems. This group of biofuels use algae as their resources for energy production.^98^ Compared to the soybeans and sunflowers original lipid content (20% and 55% respectively), the primary lipid content of algae is not considerably high (2%-40%); while its per-area-unit output of biofuel is substantially higher (>50-fold). Also, the extraction process is much more economic. The biofuel production efficiency of algae derives from its proficiency of photosynthesis, high growth rate, and the simple structure of the algae that makes its biomass much more utilizable in the biofuel making industry.^64,67,69,75^

In contrast to the plants, microalgae store their primary energy in lipid materials instead of saccharides. Microalgal primary energy storage may be in any form of fatty acids or their derivatives or any other substances and compounds that is biosynthetically or functionally related to them. Then, lipids and fatty acids are found in not only the microalgal membrane, but also as the metabolites and mere storage bodies in the cytoplasm, often located around the mitochondria. The oil molecules in microalgae are majorly found as tri-alkyl glycerides (triple long-chain fatty acids bonded to an axis of glycerol). However, isoprenoids, phospholipids, glycolipids, and hydrocarbons are also found. The microalgal lipids are mostly methyl esters which are more oxidized and more viscous compared to the crude petroleum.^64,67,69,75^

*Chlorella vulgaris* has a great potential for producing biofuel. Previously, Yoo et al showed that the maximum lipid concentration in *C. vulgaris* can reach about 11% of its final dry weight if aerated using 10% CO_2_-enriched air, putting it far after *B. braunii* that could produce oils up to about 25% of its total dry biomass.^67^ But, Widjaja et al displayed that lipid production of the *C. vulgaris* grows in the presence of further CO_2_ (~30% of its dry biomass weight). As mentioned before, it has been shown that *C. vulgaris* lipid production can also exceed 50 percent of the algae dry weight through growing in high CO_2_ levels under nitrogen-deprived condition.^64^

Based on a 2010 report, ~1% of the total cultivable land on the earth (equivalent to 14 million hectares) is under plantation for supplying resources of the producing biofuels equivalent to 1% of the global -only- “transport” fuels.^98^ While, if a given algae species produce oil as much as the 30 percent of its biomass, it can produce 1,535 kg biomass/m^2^/d, which provides enough biodiesel for the U.S oil-derived transport in a year, only on the 3% of U.S arable terrains.^22^

The heating value of algae-derives biofuel is between 40-45 MJ/kg, similar to that of fossil petroleum. Most attempts for producing biodiesel from algae after extracting the algal oil are aimed at using the same trans-esterification process as any other oilseed for producing biodiesel. Trans-esterification happens during the conversion of triglycerides in the vegetable/animal oils or free fatty acids into fatty acid alkyl esters and glycerol. This response takes place in presence of alcohol, heat, and a strong alkaline catalyst. The procedure is a well-established biodiesel production from both vegetable and algal oils.^64,67,69,75^

Anaerobic fermentation of microalgae biomass is the most simple method for producing energy from the algae due to its independency from the biomass composition.^99^ During anaerobic fermentation, the organic carbons in the biodegradable substrate are digested to the form of methane (so-called biogas) during a four-stages metabolism including 1) hydrolysis, 2) acidogenesis (producing volatile fatty acids), 3) acetogenesis (producing acetic acid from volatile fatty acids), and 4) methanogenesis (converting acetates into methane). Microalgae is among the different microorganisms contributing to biogas production with a high production efficiency.^100^ The reason is the high lipid content of the microalgae and lacking lignin compared to the conventional substrate used for biofuel production.^63^ The cell composition of microalgae significantly impacts on the yield of biofuel, since the lipids convert to oil more conveniently than proteins, and carbohydrates. Therefore, the high output of lipid and protein and the digestibility of the microalgal cell wall because of the absence of lignin bring microalgae feedstock as a highly suitable substrate producing methanolic biogas.^63^

## Conclusion

Considering the potentials of managing microalgal cell culture conformation and its fast growth, different photosynthetic algal species are addressed to be suitable resource for producing CO_2_-free nutraceuticals and biofuels. However, at present, the commercial cultivation of microalgal biomass mainly relates to a small high-value sector of industries such as food supplements or cosmetic ingredients. Up-scaling of this process needs more efforts to make the commercial microalgae production of nutraceuticals and biofuels and the required facilities should be financially justifiable. Then, more investigations are required to further improve the stability and productivity of outdoor microalgae culture systems. The systems reviewed above, help optimization of key parameters effecting on biomass, protein, antioxidant and lipid (biofuel) production efficiency. Accordingly, parameters such as light availability, temperature and nutrient composition should comprehensively regarded based on the main product of industrial facilities. However, optimization on out-door culture systems is still a remarkable limitation to address.

## Ethical Issues

Not applicable.

## Conflict of Interest

There is no conflict of interest to declare.
